# *CSF3R* Mutations Imply Adverse Prognostic Impact in Adult Acute Myeloid Leukemia Patients: A Single‐Center Retrospective Study

**DOI:** 10.1002/cam4.71471

**Published:** 2025-12-21

**Authors:** Jinjun Yang, Lian Wang, Min Zhu, Xinrong Xiang, Yu Wu, Ting Niu, Yuping Gong, Xinchuan Chen, Chuan He, Yang Dai, Xiao Shuai, Hongbing Ma

**Affiliations:** ^1^ Department of Hematology, Institute of Hematology West China Hospital, Sichuan University Chengdu P.R. China; ^2^ Department of Dermatology West China Hospital, Sichuan University Chengdu P.R. China

**Keywords:** acute myeloid leukemia, *CEBPA* bZip mutation, chemoresistance, core‐binding factor, *CSF3R*, risk stratification

## Abstract

**Background:**

Activating colony‐stimulating factor 3 receptor (*CSF3R*) mutations are uncommon in acute myeloid leukemia (AML), and their prognostic significance remains unclear.

**Methods:**

We compared clinical, treatment response, and survival data between *CSF3R*‐mutated and *CSF3R*‐wild‐type AML patients.

**Results:**

*CSF3R*‐mutated cases presented a distinct molecular profile, with T618I and T640A being the most frequent variants. Common molecular aberrations included *CEBPA bZip* mutations (40%) and CBF fusions (23%), as well as secondary‐type AML‐associated mutations such as those in *ASXL1*, *SRSF2*, and *EZH2*. In contrast, *NPM1* mutations were significantly less common in *CSF3R*‐mutated patients (*p* = 0.03). Induction responses were poorer in *CSF3R*‐mutated AML patients, with lower complete remission (55.6% vs. 78.8%, *p* = 0.004), reduced measurable residual disease (MRD) negativity (53.3% vs. 87.6%, *p* < 0.001), and shorter MRD maintenance duration (5.4 vs. 21.6 months, *p* < 0.001). According to the logistic regression analysis, *CSF3R* mutation emerged as an independent predictor of induction failure (*p* < 0.001). Survival outcomes were also inferior: the median overall survival (OS) was 20.7 months versus not reached (*p* = 0.0013), and progression‐free survival was 9.4 versus 63.6 months (*p* < 0.0001). The adverse impact of *CSF3R* mutations was most pronounced in *CEBPA* bZip AML. *CSF3R* mutations abrogated the expected favorable prognosis (3‐year OS: 44.2% vs. 88.2%, *p* = 0.0008). Conversely, *CSF3R* status did not affect outcomes in CBF‐rearranged patients (*p* = 0.70).

**Conclusions:**

*CSF3R* mutations define a distinct, high‐risk AML subset characterized by an inferior treatment response and survival, warranting incorporation into future risk stratification.

## Graphical abstract

This study aimed to elucidate the clinical and prognostic relevance of CSF3R mutations in acute myeloid leukemia (AML), with a particular focus on their molecular characteristics, treatment responses, and survival outcomes. CSF3R mutations define a distinct high‐risk subset of AML, characterized by reduced remission rates, lower measurable residual disease negativity, and significantly inferior survival—most notably among patients harboring CEBPA basic leucine zipper domain mutations.

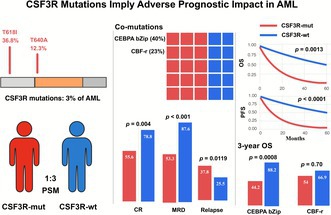

## 1 Introduction

Acute myeloid leukemia (AML) is a genetically heterogeneous malignancy in which recurrent somatic mutations critically influence prognosis and guide treatment decisions [1]. Established molecular markers such as *NPM1*, *CEBPA* bZip, *FLT3*‐ITD, and *TP53* are incorporated into the European LeukemiaNet (ELN) risk classification system [2]. However, the clinical significance of rare mutations remains unclear. One such alteration affects the colony‐stimulating factor 3 receptor gene (*CSF3R*), which encodes the granulocyte colony‐stimulating factor receptor (G‐CSF), a key regulator of granulopoiesis [3]. While activating *CSF3R* mutations are characteristic of chronic neutrophilic leukemia (CNL), they are infrequent in de novo AML, occurring in 1%–3% of cases [4]. Notably, *CSF3R* mutations in AML often cooccur with favorable‐risk lesions, including core‐binding factor (CBF) fusions and biallelic *CEBPA* mutations [5].

Recent studies have raised concerns about poorer outcomes in *CEBPA*‐mutant AML patients harboring *CSF3R* mutations; however, their broader prognostic relevance remains controversial. Some cohorts reported higher relapse rates and inferior survival [6, 7], whereas others reported no significant impact on overall survival (OS) [8, 9, 10], which is often limited by small sample sizes. Additionally, *CSF3R* mutations are implicated in leukemic transformation from severe congenital neutropenia, which is typically acquired early and followed by mutations in leukemia‐related genes such as *RUNX1*, suggesting stepwise clonal evolution [11]. These observations underscore the need to clarify the clinical impact of *CSF3R* mutations in AML.

We hypothesized that *CSF3R* mutations may identify a distinct, high‐risk AML subset with adverse outcomes independent of coexisting genetic features. To test this hypothesis, we analyzed a large single‐center cohort via propensity score matching (PSM) to minimize confounding and assess the prognostic value of *CSF3R* mutations. We further evaluated outcomes in key genomic subgroups (e.g., CBF‐rearranged, *CEBPA* bZip) and explored potential mechanisms, including comutational patterns and the influence of G‐CSF signaling.

## 2 Methods

### 2.1 Study Cohort

We retrospectively analyzed AML patients (≥ 16 years) treated at West China Hospital, Sichuan University, from January 2019 to March 2025. Among the 1897 patients who underwent next‐generation sequencing covering the full coding region of *CSF3R*, 56 (3.0%) harbored *CSF3R* mutations. After cases with incomplete data were excluded, 52 patients were included in the *CSF3R* mutation group (*CSF3R* ^mut^). To construct a control cohort, we performed 1:3 nearest‐neighbor PSM using age, the ELN 2022 risk group, and receipt of allo‐HSCT, identifying 156 *CSF3R* wild‐type (*CSF3R* ^wt^) group patients. The baseline characteristics were well balanced (Figure S1). The study was approved by the Institutional Review Board of the West China Hospital of Sichuan University (ethical approval number: 2025 Review No. 1359) and conducted in accordance with the Declaration of Helsinki. Informed consent was obtained from all patients.

### 2.2 Genomic Profiling

Targeted sequencing was performed on diagnostic bone marrow or peripheral blood samples via an Illumina platform. The panel included recurrent AML‐associated genes and the complete *CSF3R* coding region [12]. Pathogenicity was classified on the basis of the clinical significance level and grading criteria [13]. *CSF3R* mutations are categorized as membrane‐proximal activating (e.g., T618I, T615A, and T640A) or cytoplasmic truncating/frameshift variants.

### 2.3 Treatment and Response Assessment

Treatment regimens included intensive chemotherapy (e.g., “7 + 3” cytarabine plus anthracycline), venetoclax plus hypomethylating agents (VEN + HMA), or investigational VEN + HMA‐based combinations (VEN + HMA plus). The response was assessed per the Chinese AML guidelines (2017/2023) [14, 15]. Complete remission (CR) required < 5% marrow blasts, hematologic recovery, and no extramedullary disease. Partial remission (PR) was defined as 5%–25% blasts or a ≥ 50% reduction. Measurable residual disease (MRD) negativity was determined by multiparameter flow cytometry. Early death was defined as death occurring within 30 or 60 days post‐induction. The time to response and MRD negativity were measured from treatment initiation. The MRD duration was calculated from the first confirmed MRD‐negative status until reappearance, relapse, or the last follow‐up. OS was defined from diagnosis to death. Progression‐free survival (PFS) was measured from diagnosis to relapse, progression, or death. For relapsed/refractory (R/R) patients, PFS was measured from salvage initiation.

### 2.4 Statistical Analysis

Group comparisons were performed via Fisher's exact test or the chi‐square test for categorical variables and the Mann–Whitney *U* test or Student's *t* test for continuous variables. Data are presented as counts (percentages) for categorical variables and means ± SDs or medians with interquartile ranges for continuous variables, depending on their distribution. Survival probabilities were estimated via the Kaplan–Meier method and compared via the log‐rank test. Univariate logistic regression analyses were conducted to identify predictors of induction CR and MRD negativity. Variables were considered for multivariable modeling if they were clinically relevant and if *p* < 0.10 in the univariate analysis. Prior to modeling, data sparsity was assessed via the *nearZeroVar* function (caret package), and predictors with near‐zero variance or insufficient outcome counts per category were excluded. Multicollinearity was evaluated via variance inflation factors (VIFs; *car* package), with all retained covariates showing a VIF < 5. The final logistic model converges without warning. The covariates included in the multivariable model were age < 60 years, disease status (ND vs. R/R), AML‐MRC, complex karyotype, *NPM1*, *CEBPA* bZIP, *CSF3R*, *TP53*, *ASXL1*, *RUNX1*, induction therapy, and transplantation. Adjusted odds ratios (ORs) with 95% confidence intervals (CIs) are reported. All the statistical tests were two‐sided, and *p* < 0.05 was considered statistically significant. Analyses were performed via R software, version 4.4.1.

## 3 Results

### 3.1 Prevalence and Mutation Spectrum

**FIGURE 1:**
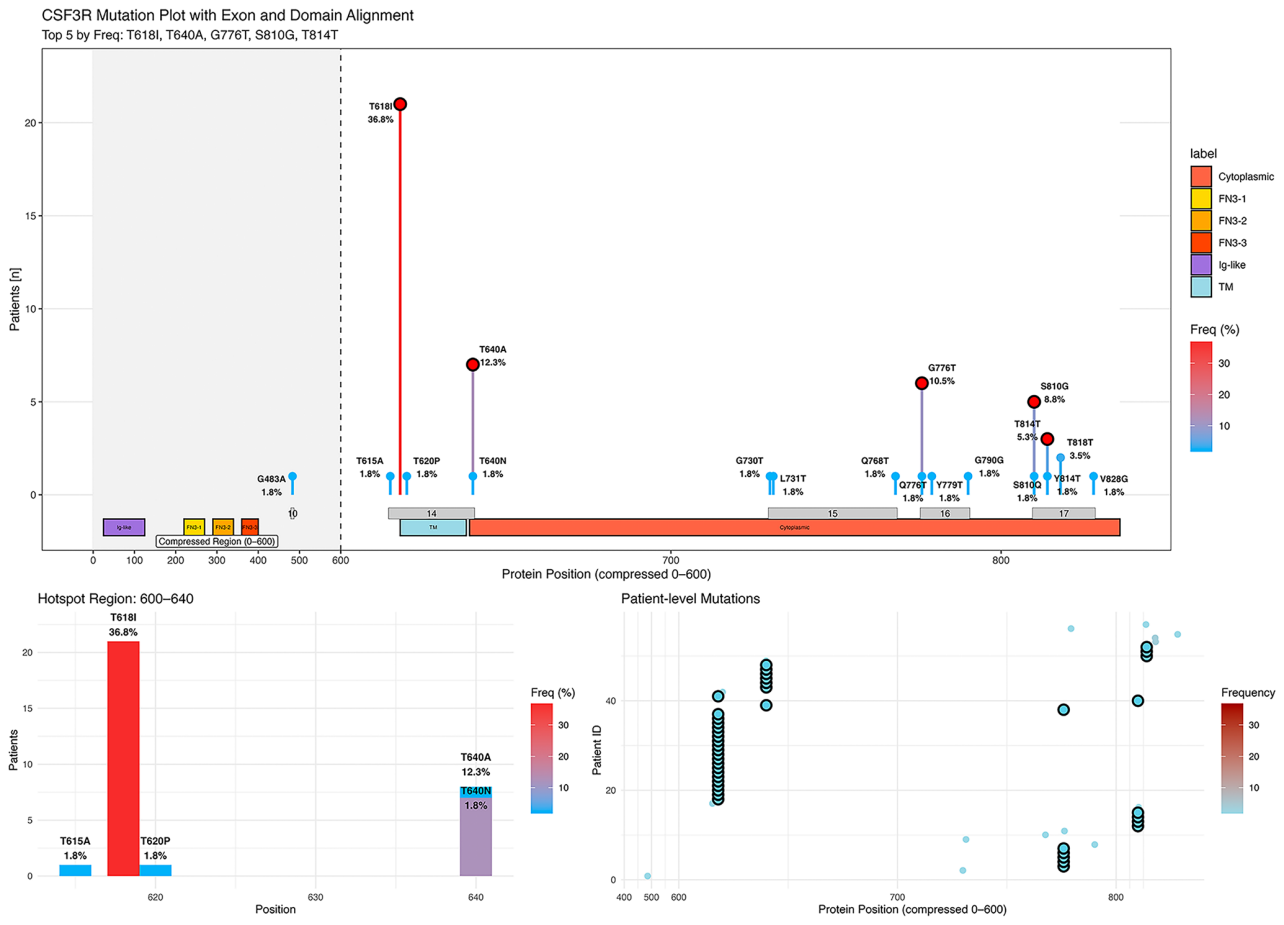
Distribution of *CSF3R* mutations in acute myeloid leukemia (AML) with exon and protein domain alignment. The top panel shows a lollipop plot of *CSF3R* mutation positions across the protein, annotated with functional domains, including immunoglobulin‐like (Ig‐like), fibronectin type III (FN3‐1, FN3‐2, FN3‐3), transmembrane (TM), and cytoplasmic regions. Mutation frequencies are indicated by both bar height and color gradient. The most frequent hotspot was T618I (36.8%), followed by T640A (12.3%), G776T (10.5%), S810G (8.8%), and T814T (5.3%). The lower panels illustrate the distribution of mutations, highlighting clustering in the extracellular region and cytoplasmic domain. The data are shown as mutation frequencies.

Among 52 *CSF3R*‐mutated AML patients, 5 (9.6%) harbored two distinct variants. *CSF3R* mutations are clustered in two hotspot regions of the receptor: the membrane‐proximal juxtamembrane domain (codons 614–640) and the cytoplasmic tail (codons 700–840). The most frequent variants were T618I (36.8%) and T640A (12.3%) in the juxtamembrane, with additional truncating or frameshift mutations (e.g., G776T, S810G, and T814T) in the tail. Overall, 56% had membrane‐proximal (exon 14) mutations, and 44% had cytoplasmic variants (exons 15–17) (Figure 1).

### 3.2 Baseline Characteristics

The clinical features are summarized in Table 1. Approximately 20%–26% of patients in each group had acute myelomonocytic or monocytic leukemia (M4/M5), with no difference in FAB subtype distribution (*p* = 0.52). *CSF3R* ^mut^ patients tended toward higher white blood cell counts (WBCs, not significant) but had significantly lower platelet counts (34 × 10^9^/L vs. 64 × 10^9^/L, *p* = 0.031), lower hemoglobin levels (7.6 vs. 8.5 g/dL, *p* = 0.027), and a lower percentage of marrow blasts (43% vs. 57%, *p* = 0.001). The trends in the R/R subgroup were less clear due to the limited sample size.

**TABLE 1:** Clinical and molecular characteristics of AML patients with *CSF3R* ^wt^ versus *CSF3R* ^mut^ status stratified by ND and R/R disease.

Characteristics	ND			R/R		
	*CSF3R* ^wt^ (*n* = 137)	*CSF3R* ^mut^ (*n* = 45)	*p*	*CSF3R* ^wt^ (*n* = 19)	*CSF3R* ^mut^ (*n* = 7)	*p*
Sex (Male)	53.0 (38.7)	24.0 (53.3)	0.121	12.0 (63.2)	5.0 (71.4)	1.000
Age (years)	46.3 (17.1)	48.8 (18.7)	0.390	43.6 (16.5)	35.9 (16.7)	0.301
AMML/AMoL	36.0 (26.3)	9.0 (20.0)	0.517	10.0 (52.6)	1.0 (14.3)	0.191
Laboratory						
WBC (×10^9^/L)	30.1 (60.2)	34.3 (51.1)	0.673	32.3 (72.3)	23.3 (38.7)	0.758
PLT (×10^9^/L)	63.6 (85.7)	34.4 (46.3)	0.031	89.6 (106.9)	62.4 (63.3)	0.536
Hb (g/dL)	8.5 (2.5)	7.6 (2.4)	0.027	9.6 (2.5)	10.5 (3.4)	0.457
PB blast (%)	36.5 (32.4)	27.0 (28.0)	0.081	22.7 (32.8)	17.0 (24.8)	0.684
BM blast (%)	56.6 (23.4)	43.3 (22.5)	0.001	34.5 (28.6)	52.4 (24.8)	0.156
Karyotype						
Complex	12.0 (8.8)	2.0 (4.4)	0.535	4.0 (21.1)	0.0 (0.0)	0.480
‐17/abn(17p)	6.0 (4.4)	0.0 (0.0)	0.344	1.0 (5.3)	0.0 (0.0)	1.000
‐5/del(5q)	4.0 (2.9)	1.0 (2.2)	1.000	1.0 (5.3)	0.0 (0.0)	1.000
‐7	4.0 (2.9)	1.0 (2.2)	1.000	1.0 (5.3)	0.0 (0.0)	1.000
Fusion gene						
CBF‐r	32.0 (23.4)	10.0 (22.2)	1.000	4.0 (21.1)	2.0 (28.6)	1.000
KMT2A‐r	4.0 (2.9)	0.0 (0.0)	0.567	1.0 (5.3)	0.0 (0.0)	1.000
EVI1	4.0 (2.9)	0.0 (0.0)	0.567	1.0 (5.3)	0.0 (0.0)	1.000
*BCR::ABL1*	3.0 (2.2)	1.0 (2.2)	1.000	NA	NA	NA
Mutation						
*NPM1*	22.0 (16.1)	1.0 (2.2)	0.030	3.0 (15.8)	0.0 (0.0)	0.670
*CEBPA* bZip	42.0 (30.7)	17.0 (37.8)	0.483	6.0 (31.6)	5.0 (71.4)	0.169
*TP53*	6.0 (4.4)	2.0 (4.4)	1.000	3.0 (15.8)	2.0 (28.6)	0.863
*ASXL1*	12.0 (8.8)	10.0 (22.2)	0.032	3.0 (15.8)	0.0 (0.0)	0.670
*PTPN11*	14.0 (10.2)	5.0 (11.1)	1.000	3.0 (15.8)	0.0 (0.0)	0.670
*RUNX1*	11.0 (8.0)	7.0 (15.6)	0.238	1.0 (5.3)	0.0 (0.0)	1.000
*RAS*	23.0 (16.8)	12.0 (26.7)	0.215	2.0 (10.5)	0.0 (0.0)	0.949
*IDH*	22.0 (16.1)	4.0 (8.9)	0.344	1.0 (5.3)	2.0 (28.6)	0.338
*KIT*	16.0 (11.7)	8.0 (17.8)	0.426	1.0 (5.3)	0.0 (0.0)	1.000
*FLT3‐*ITD	18.0 (13.1)	3.0 (6.7)	0.363	3.0 (15.8)	1.0 (14.3)	1.000
*U2AF1*	4.0 (2.9)	1.0 (2.2)	1.000	NA	NA	NA
*SRSF2*	2.0 (1.5)	5.0 (11.1)	0.013	NA	NA	NA
*STAG2*	6.0 (4.4)	3.0 (6.7)	0.828	1.0 (5.3)	0.0 (0.0)	1.000
*BCOR*	4.0 (2.9)	5.0 (11.1)	0.071	NA	NA	NA
*ZRSR2*	0.0 (0.0)	2.0 (4.4)	0.097	NA	NA	NA
*EZH2*	0.0 (0.0)	5.0 (11.1)	0.001	NA	NA	NA
Treatment			0.149			0.168
CT	95.0 (69.3)	25.0 (55.6)		9.0 (47.4)	5.0 (71.4)	
VEN + HMA	18.0 (13.1)	11.0 (24.4)		3.0 (15.8)	2.0 (28.6)	
VEN + HMA plus	24.0 (17.5)	9.0 (20.0)		7.0 (36.8)	0.0 (0.0)	
Transplantation	21.0 (15.3)	8.0 (17.8)	0.877	2.0 (10.5)	0.0 (0.0)	0.949

*Note:* Data are shown as counts (percentages) for categorical variables and means (±SDs) or medians with interquartile ranges for continuous variables, depending on their distribution. *p* values compare *CSF3R* ^wt^ versus *CSF3R* ^mut^ within the ND and R/R cohorts, respectively.

Abbreviations: AML, acute myeloid leukemia; AMML/AMoL, acute myelomonocytic or monocytic leukemia; CBF, core‐binding factor; HMA, hypomethylating agent; KMT2A, mixed‐lineage leukemia gene; NA, not applicable; ND, newly diagnosed; R/R, relapsed or refractory; VEN, venetoclax.

### 3.3 Molecular Landscape

**FIGURE 2:**
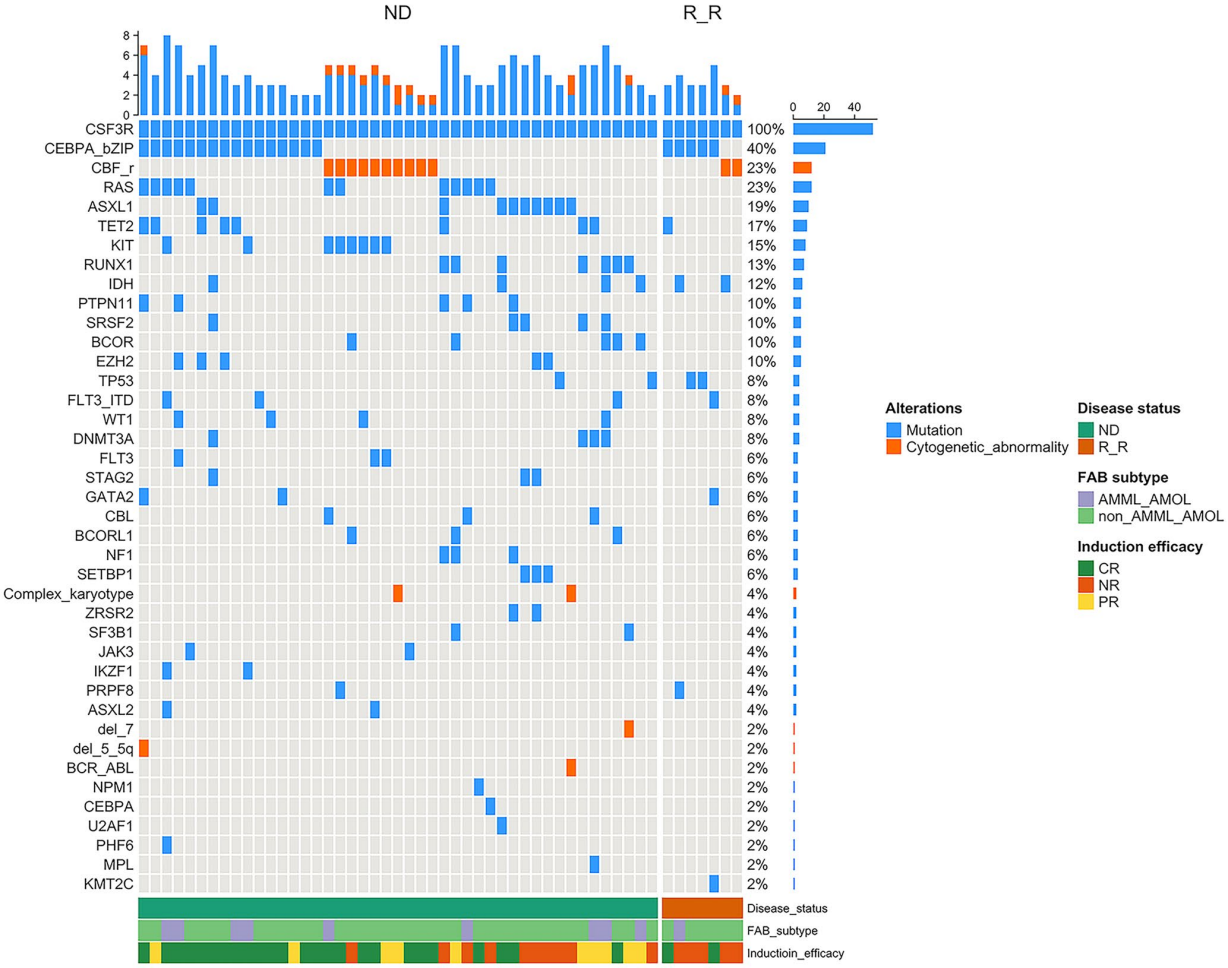
Genomic and cytogenetic landscape of *CSF3R*‐mutated AML stratified by disease phase. Each column represents a *CSF3R*‐mutated patient, grouped by newly diagnosed (ND) or relapsed/refractory (R/R) status. Each row represents a gene mutation or cytogenetic abnormality. The blue squares indicate gene mutations; the orange squares indicate cytogenetic events. The top bar denotes the per‐patient alteration count, and the side bar denotes the alteration frequency. Frequent comutations included *CEBPA* bZip (40%), core‐binding factor rearrangements (CBF‐r, 23%), and *RAS* (23%). Data represent binary mutation status (presence/absence) for each gene or cytogenetic event (*n* = 52 *CSF3R*‐mutated AML samples).

All *CSF3R* ^mut^ patients carried at least one additional somatic or cytogenetic alteration. Comutations commonly included *CEBPA* bZip (40%, mostly biallelic) and CBF fusions (23%) (Figure 2). *CSF3R* ^mut^ AML patients presented higher frequencies of mutations typical of secondary or myelodysplasia‐related AML. *ASXL1* mutations were more common in *CSF3R* ^mut^ patients (22.2% vs. 8.8%, *p* = 0.032), as were *SRSF2* (11.1% vs. 1.5%, *p* = 0.013) and EZH2 (11.1% vs. 0%, *p* = 0.001) mutations. Conversely, *NPM1* mutations were less common in *CSF3R* ^mut^ patients (2.2% vs. 16.1%, *p* = 0.03). Moreover, mutations in genes such as *FLT3*‐ITD, *RUNX1*, *BCOR*, *IDH1/2*, *KRAS*/*NRAS*, *WT1*, and *TET2* occurred at similar modest frequencies in both groups (Table 1).

### 3.4 Induction Response

**FIGURE 3:**
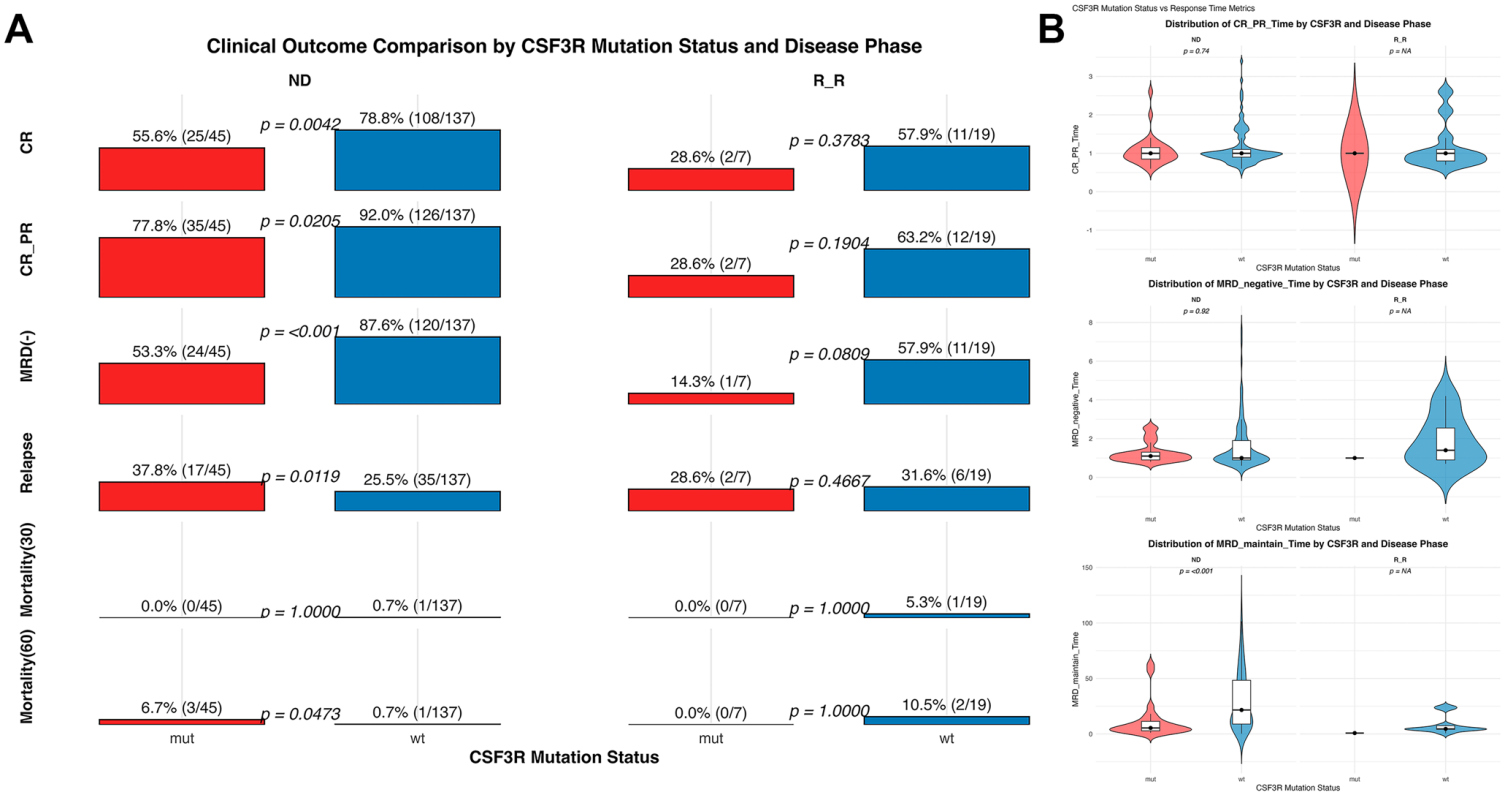
Clinical outcomes and response kinetics in AML patients stratified by *CSF3R* mutation status. (A) Bar plots comparing the CR, CR + PR, MRD negativity, relapse, and early mortality (30/60 days) rates between *CSF3R*‐mutated (mut) and wild‐type (wt) AML patients across the ND and R/R cohorts. The percentages and sample sizes are shown above each bar. (B) Violin plots showing the distribution of response‐related time intervals, including time to CR/PR, time to MRD negativity, and MRD maintenance duration, stratified by *CSF3R* mutation and disease status (ND vs. R/R).

Among ND patients receiving intensive chemotherapy, *CSF3R* ^mut^ patients had significantly lower CR rates (55.6% vs. 78.8%, *p* = 0.0042) and overall response rates (77.8% vs. 92.0%, *p* = 0.021). MRD negativity was notably reduced (53.3% vs. 87.6%, *p* < 0.001), and early relapse was more common (37.8% vs. 25.5%, *p* = 0.012). Although 30‐day mortality was 0% in *CSF3R* ^mut^ patients versus 0.7% in wild‐type patients, 60‐day mortality was higher (6.7% vs. 0.7%, *p* = 0.047), largely due to refractory disease with infection. In the R/R cohort (*n* = 26), *CSF3R* ^mut^ patients had lower CR rates (28.6% vs. 57.9%) and MRD negativity rates (14.3% vs. 57.9%) and greater rates of primary refractory disease (71.4% vs. 36.8%), although these differences were not statistically significant (*p* = 0.378, *p* = 0.081, and *p* = 0.190, respectively) (Figure 3A).

To further explore the impact of *CSF3R* mutations on treatment kinetics, we analyzed time–response metrics stratified by disease status. Among ND patients, the median time to CR or PR was similar between the groups (*p* = 0.74). Similarly, the time to MRD negativity did not significantly differ (*p* = 0.92). However, MRD maintenance was significantly shorter in *CSF3R* ^mut^ patients (median 5.4 vs. 21.6 months, *p* < 0.001) (Figure 3B). The limited sample size precluded reliable comparisons in the R/R subgroup.

### 3.5 Predictors of Induction Failure: *CSF3R* As an Independent Adverse Factor

**FIGURE 4:**
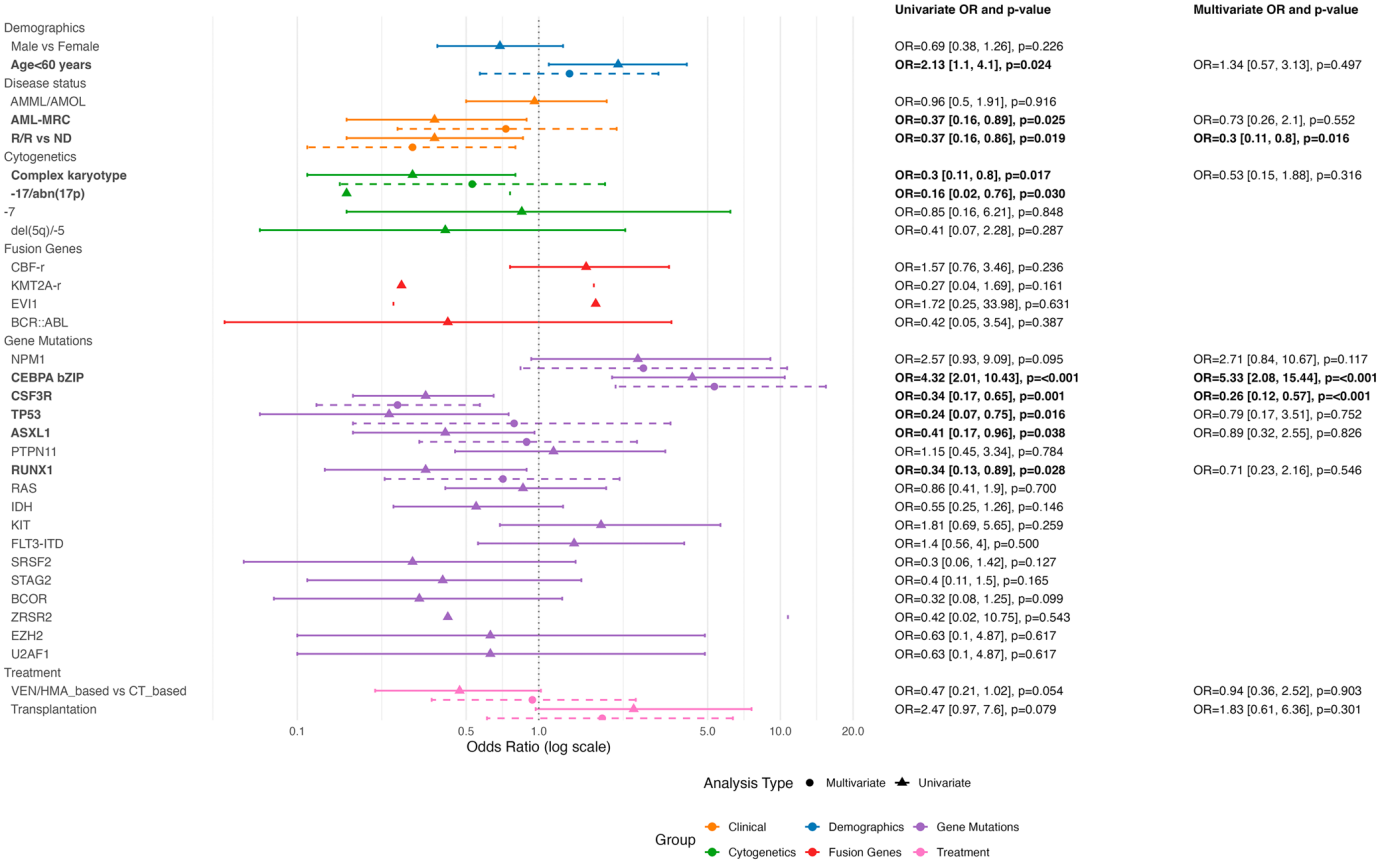
Forest plot showing predictors of complete remission (CR) in patients with AML. Odds ratios (ORs) and 95% confidence intervals (CIs) from univariate (triangles) and multivariate (circles) logistic regression analyses are plotted on a logarithmic scale. Variable groups are color‐coded: Demographics (blue), clinical (orange), cytogenetic (green), fusion genes (red), gene mutations (purple), and treatment (pink). In multivariate analysis, *CSF3R* mutation and relapse/refractory status were associated with significantly lower odds of achieving CR (OR = 0.26, 95% CI 0.12–0.57, *p* = 0.001; OR = 0.3, 95% CI 0.11–0.8, *p* = 0.016), whereas *CEBPA* bZip mutation predicted a favorable response (OR = 5.33, 95% CI 2.08–15.44, *p* = 0.001). Only variables meeting the inclusion criteria based on univariate screening (*p* < 0.10) and data adequacy were entered into the multivariate model. Two‐sided *p* values were calculated from logistic regression models (*n* = 208).

Univariate logistic regression identified *CSF3R* mutation as a strong negative predictor of CR (OR 0.34; 95% CI, 0.17–0.65; *p* = 0.001), along with myelodysplasia‐related changes (AML‐MRC), R/R status, *TP53*, *ASXL1*, *RUNX1*, monosomy 17 or 17p abnormalities, and complex karyotypes. Favorable factors included younger age and *CEBPA* bZip. Interestingly, *CBF fusions* and *NPM1* did not significantly influence CR in this matched cohort (Figure 4). According to the multivariate analysis, *CSF3R* mutation was independently associated with induction failure (adjusted OR 0.26; 95% CI, 0.12–0.57; *p* < 0.001), as was R/R disease (OR 0.3; *p* = 0.016). The *CEBPA* bZip mutation had a strong positive impact (OR 5.33; *p* < 0.001). A sensitivity analysis excluding R/R patients yielded similar findings (*CSF3R* ^mut^ OR 0.27; *p* < 0.001), reinforcing its prognostic value in de novo AML (Figure S2).

For MRD‐negative patients, univariate analysis suggested that *CSF3R* ^mut^ also independently predicted failure (OR 0.18; 95% CI, 0.09–0.35; *p* < 0.001). Other adverse predictors included male sex, AML‐MRC, R/R disease, *TP53*, *ASXL1*, *RUNX1*, and *SRSF2*, monosomy 17 or 17p abnormalities, complex karyotypes, and the use of VEN + HMA‐based regimens. The favorable factors were younger age and CBF fusion. In the multivariable model, *CSF3R* mutation retained its independent negative prognostic value (adjusted OR 0.08; 95% CI, 0.03–0.21; *p* < 0.001) (Figure S3). A sensitivity analysis excluding R/R patients yielded similar findings (*CSF3R* ^mut^ OR 0.1; *p* < 0.001) (Figure S4).

### 3.6 Survival Outcomes

**FIGURE 5:**
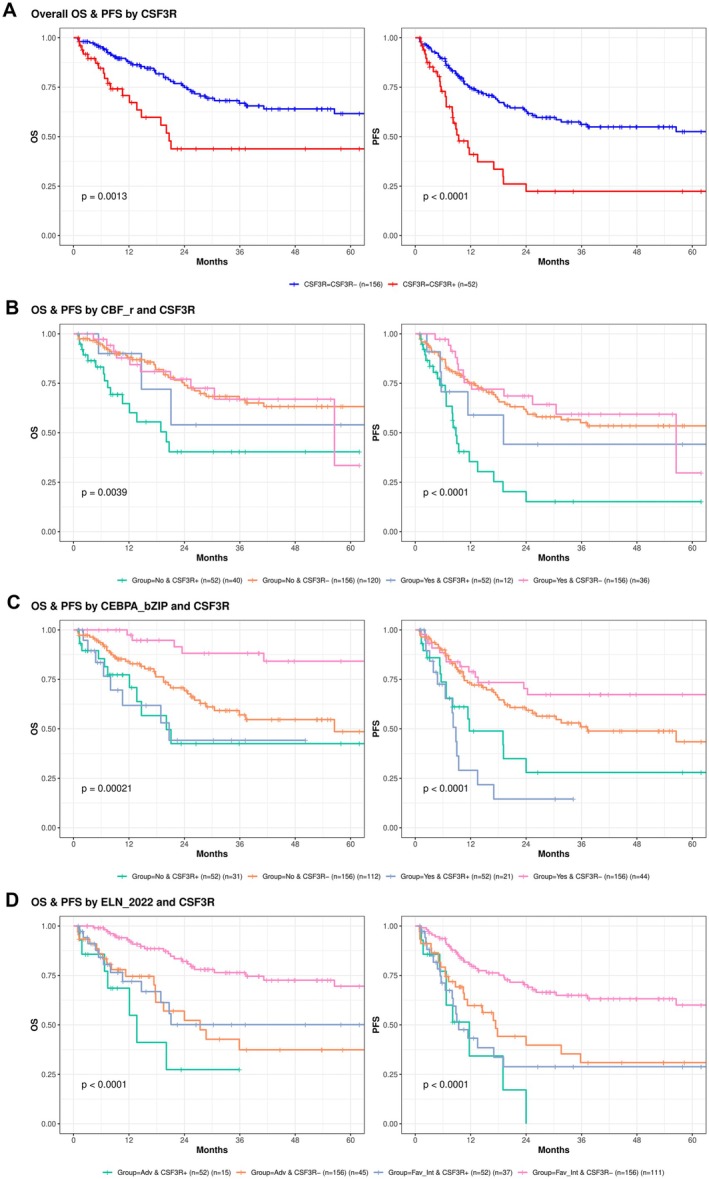
Kaplan–Meier analysis of overall survival (OS) and progression‐free survival (PFS) according to *CSF3R* mutation status and clinical subgroup. (A) OS and PFS curves for *CSF3R*‐mutated versus wild‐type AML patients. (B) OS/PFS stratified by CBF rearrangement. (C) OS/PFS stratified by *CEBPA* bZip mutation. (D) OS/PFS stratified by ELN 2022 risk classification. In all the comparisons, *CSF3R*‐mutated patients presented significantly inferior OS and PFS. *p* values were calculated via the two‐sided log‐rank test. The data are shown as Kaplan–Meier estimates with 95% CIs; tick marks indicate censored observations (*n* = 208).

At the 29‐month median follow‐up, *CSF3R* mutations were associated with inferior OS and PFS (Figure 5). The median OS was 20.7 months for *CSF3R* ^mut^ patients but was not reached in the *CSF3R* ^wt^ group (*p* = 0.0013) (Figure 5A). The 3‐year OS rate was 43.8% in the *CSF3R* ^mut^ group and 66.9% in the *CSF3R* ^wt^ group (*p* = 0.0022) (Table S1). The median PFS was 9.4 months in the *CSF3R* ^mut^ group and 63.6 months in the *CSF3R* ^wt^ group (*p* < 0.0001) (Figure 5A), and the 3‐year PFS rate was 22.4% for *CSF3R* ^mut^ patients and 56.2% for *CSF3R* ^wt^ patients (*p* = 0.001) (Table S1).

### 3.7 Subtype Analyses

In CBF‐rearranged AML (*n* = 48), *CSF3R* mutations were associated with a nonsignificant trend toward worse survival (3‐year OS: 54.0% vs. 66.9%, *p* = 0.700; 3‐year PFS: 44.2% vs. 59.4%, *p* = 0.700; respectively) (Table S2). In contrast, among non‐CBF‐rearranged AML patients, *CSF3R* ^mut^ patients had markedly worse median OS and PFS (OS: 20.1 vs. not reached, *p* = 0.0007; PFS: 8.8 vs. not reached, *p* < 0.0001) (Figure 5B). The 3‐year OS rates were 40.3% and 66.7% (*p* = 0.0022), and the 3‐year PFS rates were 15.2% and 55.1% (*p* = 0.0008) (Table S2).

Among 65 *CEBPA* bZip AML patients, *CSF3R* mutations abrogated the favorable prognosis. The median OS was 20.7 months for *CSF3R* ^mut^ patients but not for *CSF3R* ^wt^ patients (*p* < 0.0001) (Figure 5C), and the 3‐year OS rates were 44.2% and 88.2%, respectively (*p* = 0.0008) (Table S3). The median PFS was 8.8 months versus not reached (*p* < 0.0001) (Figure 5C), and the 3‐year PFS was 0.0% versus 67.3% (*p* = 0.0006) (Table S3). Among non‐*CEBPA* bZip patients, *CSF3R* mutation also had a negative effect on 1‐year OS (42.5% vs. 57.0%, *p* = 0.0149) and 1‐year PFS (27.9% vs. 59.4%, *p* = 0.035) (data not shown).

In the ELN 2022 favorable/intermediate‐risk group, *CSF3R* mutations conferred inferior outcomes. The median OS was not reached in either group (*p* = 0.0018) (Figure 5D), and the 3‐year OS rates were 50.1% and 76.4% (*p* = 0.0065) (Table S4). In the adverse‐risk group, those harboring *CSF3R* mutations represented a particularly high‐risk subset, with no patients (0/15) surviving beyond 3 years. The median OS was 13.7 months in *CSF3R* ^mut^ patients compared with 27.4 months in *CSF3R* ^wt^ patients, although this difference was not statistically significant because of the limited sample size. PFS trends mirrored those of OS (Figure 5D).

## 4 Discussion

Our study suggests that *CSF3R* mutations may define a high‐risk subset of adult AML. Using a large PSM cohort, we showed that *CSF3R* ^mut^ AML has unique molecular associations and significantly worse clinical outcomes than *CSF3R* ^wt^ AML does. Importantly, we demonstrated that *CSF3R* mutations confer an adverse prognosis independent of other risk factors and particularly exacerbate the risk of otherwise favorable‐genetic AML. Below, we highlight and interpret our major findings and discuss their implications for AML biology and management.

First, we found that *CSF3R*‐mutated AML displays a characteristic genetic landscape. The association between *CSF3R* mutations and alterations in transcription factors, particularly *CEBPA* and CBF fusions, has been well established. Zhang et al. reported that 90.5% of *CSF3R* ^mut^ AML cases harbored either CBF fusions or biallelic *CEBPA* mutations [5]. Similarly, Chen et al. identified *RUNX1::RUNX1T1*, *CEBPA*, and *TET2* as the most frequent comutations in *CSF3R* ^mut^ AML in a Chinese population [8]. Consistent with these findings, we observed *CEBPA* bZip mutations in 40% and CBF fusions in 23% of our *CSF3R* ^mut^ cases. Interestingly, our data differed from those of Zhang et al. with respect to *NPM1*. While they reported an association between *CSF3R* and *NPM1* mutations [5], we found that *CSF3R* mutations were markedly underrepresented in *NPM1*‐mutated AML. This discrepancy may reflect biological differences or cohort‐specific selection biases. In addition, a second molecular context was evident: *CSF3R* ^mut^ patients frequently harbored mutations typical of AML with myelodysplasia‐related changes, such as those in *ASXL1* and *SRSF2*. These findings support the notion that *CSF3R*‐driven leukemias may originate from two distinct biological settings: (1) de novo AML with otherwise favorable lesions and (2) secondary AML evolving from antecedent myeloid neoplasms. This duality is consistent with clinical observations. Some patients had a history of CNL or atypical chronic myeloid leukemia, whereas others presented de novo with CBF or *CEBPA* alterations. The bimodal molecular landscape of *CSF3R* ^mut^ AML suggests divergent leukemogenic pathways that merit further genomic and functional investigation. Regardless of origin, a unifying feature is that *CSF3R* mutations rarely occur in isolation. All *CSF3R* ^mut^ patients in our cohort harbored at least one additional pathogenic mutation or cytogenetic abnormality, underscoring the role of *CSF3R* as a cooperative lesion within a broader mutational framework in AML.

Second, our data provide clear evidence that *CSF3R* mutations are associated with a myeloproliferative clinical phenotype. At diagnosis, *CSF3R* ^mut^ patients tend to have high WBC counts but, paradoxically, lower marrow blast percentages, although the disease has a chronic proliferative component. This parallels CNL biology (where *CSF3R* T618I drives neutrophilic proliferation) and could indicate that *CSF3R* signaling biases blasts toward partial differentiation. Clinically, this might manifest as leukocytosis with fewer blasts—a clue that should prompt testing for *CSF3R* in such AML presentations. These findings are consistent with those reported by Su et al., who reported that patients with *CSF3R* mutations had significantly lower platelet counts and higher leukocyte counts than their *CSF3R* wild‐type counterparts did [7].

Third, and more importantly, we observed a significantly poorer response to induction therapy in patients with *CSF3R*‐mutated AML. The CR rate was approximately 30% lower than that of matched controls, and among those who achieved CR, MRD positivity was frequently observed. Multivariate analysis revealed *CSF3R* mutation as one of the strongest independent predictors of induction failure. This finding is novel for adult AML patients and underscores that *CSF3R* mutations confer a chemotherapy‐refractory phenotype. One possible explanation is that *CSF3R* activation promotes differentiation toward the neutrophil lineage—as observed in CNL—thereby enabling leukemic cells to exit the highly proliferative blast state most susceptible to cytotoxic agents. As a result, these cells may evade eradication by conventional chemotherapy. Additionally, sustained *CSF3R* signaling via the JAK–STAT5 and SRC pathways may activate prosurvival signaling cascades, reducing apoptosis under genotoxic stress [16]. The high co‐occurrence of *ASXL1* and *SRSF2* mutations, which are known to confer resistance to apoptosis and DNA damage [17, 18], likely amplifies this effect. Our hypothesis is that these mutations synergize to create a clone that is both proliferative and resilient to DNA‐damaging agents—a “perfect storm” for chemoresistance. This is supported by our observation that *CSF3R* ^mut^ patients who failed induction often had multiple such adverse comutations, whereas those who achieved CR tended to lack them. Nevertheless, even after accounting for comutations, *CSF3R* itself remains an independent culprit. This finding suggests a direct effect of constitutive *CSF3R* signaling on leukemia cell biology (e.g., promoting the survival of a more differentiated, chemotherapy‐insensitive population of leukemic cells). Notably, in CNL, membrane‐proximal *CSF3R* mutations (such as T618I) are associated with aggressive disease, whereas truncation mutations might be less proliferative [4]. We did not observe an obvious difference in outcome according to *CSF3R* mutation type in our AML cohort (likely due to limited numbers per subtype), but it remains possible that, for example, T618I versus G776T could have different impacts. Larger studies could address this issue.

Fourth, previous studies have suggested that *CSF3R* mutations adversely impact PFS in *CEBPA* double‐mutant AML; however, their prognostic significance for OS in AML patients remains controversial [6, 7, 8, 9, 10]. Our findings clearly demonstrate that *CSF3R* mutations are associated with poor survival outcomes. Notably, some long‐term survivors were present, typically those who underwent allo‐HSCT or achieved CR after multiple lines of therapy. However, the majority of *CSF3R*‐mutated patients die early due to refractory disease or relapse. Importantly, these survival differences persisted despite a similar proportion of patients receiving allo‐HSCT in both groups. This observation underscores the need for early identification of high‐risk patients and consideration of more intensive or novel therapeutic strategies in *CSF3R*‐mutated AML. Similarly, Cao et al. reported that *CSF3R* mutation status did not significantly affect 3‐year survival or relapse rates in de novo AML patients following allo‐HSCT [19].

Finally, one of the most clinically impactful findings of our study is the adverse prognostic interaction between *CSF3R* mutations and other risk factors. In patients classified as low risk by current criteria—such as those with *CEBPA* bZip mutations—the presence of a *CSF3R* mutation was associated with markedly worse outcomes, effectively reclassifying them into a high‐risk category. For example, a *CSF3R*‐mutated *CEBPA* bZip AML patient in our series had a prognosis akin to that of an adverse‐risk AML patient (with early relapse and death), whereas a similar patient without a *CSF3R* mutation would likely be cured with chemotherapy alone. This highlights a critical gap in existing risk models, such as ELN 2022, which do not currently account for *CSF3R* status. Our data suggest that patients deemed “favorable risk” on the basis solely of the *CEBPA* marker may be inaccurately stratified if *CSF3R* mutations are present. Mechanistically, this may reflect the synergy between *CSF3R*‐driven proliferation and differentiation arrest caused by *CEBPA* mutations, resulting in a chemoresistant leukemic phenotype. Prior research noted recurring *CSF3R* mutations in *CEBPA*‐mutant AML and sensitivity to JAK inhibitors, supporting this idea [20]. Similarly, the coexistence of *CSF3R* with CBF fusions might have special significance, as suggested by the small patient numbers [9]. One study reported that pediatric CBF fusion AML patients with both *CSF3R* and *KIT* mutations had worse outcomes than those with either mutation alone did, which aligns with our observation that targeting *KIT* might have helped some of our CBF‐rearranged/*CSF3R* patients [21]. Across our cohort, *CSF3R* mutations were associated with poor outcomes even among patients with favorable or intermediate‐risk ELNs, and an ultrahigh‐risk subset was identified among those with adverse ELN risk. These findings support the incorporation of *CSF3R* mutation status into future AML risk stratification models, potentially suggesting its classification as a high‐risk marker, particularly in the presence of otherwise favorable genetic features.

On the basis of our findings, we recommend that clinicians consider intensifying therapy if a *CSF3R* mutation is detected. For fit patients, this could mean proceeding to allogeneic transplant in first remission (even if they have a normally “favorable” genotype such as *CEBPA* bZip). For patients deemed unfit for intensive therapy, early consideration of clinical trial enrollment or novel targeted agents is warranted, as standard regimens often prove insufficient. A promising therapeutic avenue involves targeting the downstream signaling of *CSF3R*. Although no direct *CSF3R* inhibitors are currently available, the receptor signals primarily through the JAK–STAT pathway. Oncogenic *CSF3R* mutations, such as T618I, T615A, and T640N, promote ligand‐independent receptor dimerization and constitutive activation of JAK–STAT signaling [22]. Preclinical studies have demonstrated that mutant *CSF3R* confers a clonal advantage at the hematopoietic stem cell level, enhancing G‐CSF–induced proliferation and increasing STAT5 activity [23]. Notably, the JAK1/2 inhibitor ruxolitinib has shown efficacy in patients with *CSF3R* T618I‐positive CNL and anecdotal benefit in other *CSF3R*‐mutated myeloid neoplasms [24]. In vitro data further support ruxolitinib sensitivity in *CSF3R*‐mutated malignancies [25]. These findings provide a strong rationale for investigating JAK inhibition in *CSF3R*‐mutated AML. A potential therapeutic strategy may involve incorporating ruxolitinib into induction or consolidation regimens to suppress *CSF3R*‐driven proliferative clones and improve clinical outcomes in this high‐risk subgroup.

We acknowledge several limitations, including the retrospective nature, modest sample size (*n* = 52), and single‐center scope. Although PSM was applied to mitigate selection bias, residual confounding cannot be completely excluded. External validation in larger, multicenter cohorts is warranted. Moreover, our analysis focused exclusively on adult patients, and pediatric AML patients may exhibit distinct biological and clinical characteristics [6].

## 5 Conclusions

In conclusion, our results establish that *CSF3R* mutation is a powerful adverse prognostic factor in AML patients. *CSF3R*‐mutated AML patients have distinctive comutations, respond poorly to standard therapies, and have significantly shorter remission and survival. These effects are independent of other risk factors and are especially notable in patients who otherwise would be considered to have a lower risk. Our findings strongly support incorporating *CSF3R* status into AML risk stratification models and clinical decision‐making. For patients with *CSF3R*‐mutated AML, clinicians should increase treatment intensity and consider novel approaches, as conventional chemotherapy is often insufficient. We also advocate exploring targeted therapeutic interventions, particularly JAK–STAT pathway inhibitors, in this patient subset, potentially through clinical trials. By recognizing *CSF3R* mutations as a marker of high‐risk disease, we can better identify patients in need of aggressive or innovative treatment, hopefully improving outcomes for this challenging subset of AML.

## Author Contributions

**Jinjun Yang:** writing – original draft (lead). **Lian Wang:** writing – original draft (supporting). **Min Zhu:** methodology (equal), software (equal). **Xinrong Xiang:** methodology (equal), software (equal). **Yu Wu:** resources (equal). **Ting Niu:** resources (equal). **Yuping Gong:** resources (equal). **Xinchuan Chen:** resources (equal). **Chuan He:** resources (equal). **Yang Dai:** resources (equal). **Xiao Shuai:** resources (equal). **Hongbing Ma:** resources (equal), writing – review and editing (lead).

## Funding

This study was supported by the General Program of the Natural Science Foundation of Sichuan Provincial Department of Science and Technology (2025ZNSFSC0567 to Hongbing Ma) and the 1·3·5 Project for Disciplines of Excellence–Clinical Research Fund, West China Hospital, Sichuan University (2025HXFH025 to Jinjun Yang).

## Ethics Statement

This study was conducted in compliance with the ethical principles of the Declaration of Helsinki and was approved by the Institutional Review Board of West China Hospital, Sichuan University (Ethical approval number: 2025 Review No. 1359).

## Consent

All patients provided written informed consent for the collection and use of their clinical data.

## Conflicts of Interest

The authors declare no conflicts of interest.

## Supporting information


**Data S1:** Supplementary Information.

## Data Availability

The datasets generated and/or analyzed during the current study are available from the corresponding author upon reasonable request.
