# Supplementary material for: CSF3R Mutations Imply Adverse Prognostic Impact in Adult Acute Myeloid Leukemia Patients: A Single‐Center Retrospective Study
Source: Cancer Med. 2025 Dec 21;14(24):e71471. doi: 10.1002/cam4.71471 (PMC12719048; doi:10.1002/cam4.71471)
Supplement: Supplementary file 1 — Data S1: Supplementary Information. [file CAM4-14-e71471-s001.docx]

**Supplementary materials**

Table S1. Comparison of survival outcomes between *CSF3R*^mut^ and *CSF3R*^wt^ in overall AML cohort.

| Outcomes | Overall | | |
| --- | --- | --- | --- |
|  | *CSF3R*^mut^ | *CSF3R*^wt^ | *p* |
| 3-year OS (%) | 43.8 | 66.9 | 0.0022 |
| 3-year PFS (%) | 22.4 | 56.2 | 0.0010 |

Abbreviations: OS, overall survival; PFS, progression-free survival; *CSF3R*^mut^, *CSF3R* mutations; *CSF3R*^wt^, *CSF3R* wild type; AML, acute myeloid leukemia.

Table S2. Comparison of survival outcomes between *CSF3R*^mut^ and *CSF3R*^wt^ in CBF-rearranged and non-CBF AML.

| Outcomes | CBF-r | | | non-CBF-r | | |
| --- | --- | --- | --- | --- | --- | --- |
|  | *CSF3R*^mut^ | *CSF3R*^wt^ | *p* | *CSF3R*^mut^ | *CSF3R*^wt^ | *p* |
| 3-year OS (%) | 54 | 66.9 | 0.700 | 40.3 | 66.7 | 0.0022 |
| 3-year PFS (%) | 44.2 | 59.4 | 0.700 | 15.2 | 55.1 | 0.0008 |

Abbreviations: OS, overall survival; PFS, progression-free survival; CBF, core-binding factor; *CSF3R*^mut^, *CSF3R* mutations; *CSF3R*^wt^, *CSF3R* wild type; AML, acute myeloid leukemia.

Table S3. Comparison of survival outcomes between *CSF3R*^mut^ and *CSF3R*^wt^ in *CEBPA* bZip and non-*CEBPA* bZip AML.

| Outcomes | *CEBPA* bZip | | | non-*CEBPA* bZip | | |
| --- | --- | --- | --- | --- | --- | --- |
|  | *CSF3R*^mut^ | *CSF3R*^wt^ | *p* | *CSF3R*^mut^ | *CSF3R*^wt^ | *p* |
| 3-year OS (%) | 44.2 | 88.2 | 0.0008 | 42.5 | 57.0 | 0.160 |
| 3-year PFS (%) | 0 | 67.3 | 0.0006 | 27.9 | 51.0 | 0.160 |

Abbreviations: OS, overall survival; PFS, progression-free survival; bZip, basic leucine zipper domain; *CSF3R*^mut^, *CSF3R* mutations; *CSF3R*^wt^, *CSF3R* wild type; AML, acute myeloid leukemia.

Table S4. Comparison of survival outcomes between *CSF3R*^mut^ and *CSF3R*^wt^ in ELN 2022 favorable/intermediate and adverse group AML.

| Outcomes | Favorable/intermediate | | | Adverse | | |
| --- | --- | --- | --- | --- | --- | --- |
|  | *CSF3R*^mut^ | *CSF3R*^wt^ | *p* | *CSF3R*^mut^ | *CSF3R*^wt^ | *p* |
| 3-year OS (%) | 50.1 | 76.4 | 0.0065 | 0.0 | 37.4 | 0.246 |
| 3-year PFS (%) | 28.8 | 65.0 | 0.0032 | 0.0 | 30.9 | 0.246 |

Abbreviations: OS, overall survival; PFS, progression-free survival; *CSF3R*^mut^, *CSF3R* mutations; *CSF3R*^wt^, *CSF3R* wild type; AML, acute myeloid leukemia.

# Figure Legends

**Figure S1. Propensity score distribution before and after matching.** Histograms show estimated propensity scores for treated and control groups before and after 1:3 nearest-neighbor matching. Post-matching distributions indicate improved balance between groups.

**Figure S2. Forest plot showing predictors of complete remission (CR) in patients with newly diagnosed acute myeloid leukemia (AML).** Odds ratios (ORs) and 95% confidence intervals (CIs) from univariate (triangles) and multivariate (circles) logistic regression analyses are plotted on a logarithmic scale. Variable groups are color-coded: demographics (blue), clinical (orange), cytogenetic (green), fusion genes (red), gene mutations (purple), and treatment (pink). In multivariate analysis, *CSF3R* mutation independently predicted a lower likelihood of achieving CR (OR = 0.27, 95% CI 0.12‒0.58, *p* = 0.001), whereas *CEBPA* bZip mutation predicted a favorable response (OR = 4.59, 95% CI 1.86‒12.6, *p* = 0.002). Only variables meeting inclusion criteria based on univariate screening (*p* < 0.10) and adequate event counts were entered into the multivariate model. Two-sided *p* values were calculated from logistic regression models (n = 208).

**Figure S3. Forest plot showing predictors of measurable residual disease (MRD) negativity in AML.** *CSF3R* mutation, relapsed/refractory disease, and complex karyotype independently predicted reduced MRD clearance (OR = 0.08, 95% CI 0.03‒0.21, *p* = 0.001; OR = 0.14, 95% CI 0.04‒0.4, *p* = 0.001; OR = 0.17, 95% CI 0.04‒0.84, *p* = 0.027). In contrast, *CEBPA* bZip mutation was associated with increased odds of achieving MRD negativity (OR = 4, 95% CI 1.3‒13.62, *p* = 0.02).

**Figure S4. Forest plot showing predictors of MRD negativity in patients with newly diagnosed AML.** *CSF3R* mutation (OR = 0.1, 95% CI 0.04‒0.25, *p* = 0.001) and complex karyotype (OR = 0.21, 95% CI 0.05‒0.99, *p* = 0.044) independently predicted lower odds of achieving MRD negativity, whereas *CEBPA* bZip mutation (OR = 3.09, 95% CI 1.06‒9.87, *p* = 0.046) and CBF rearrangement (OR = 4.25, 95% CI 1.17‒18.72, *p* = 0.038) were associated with higher odds of MRD clearance.


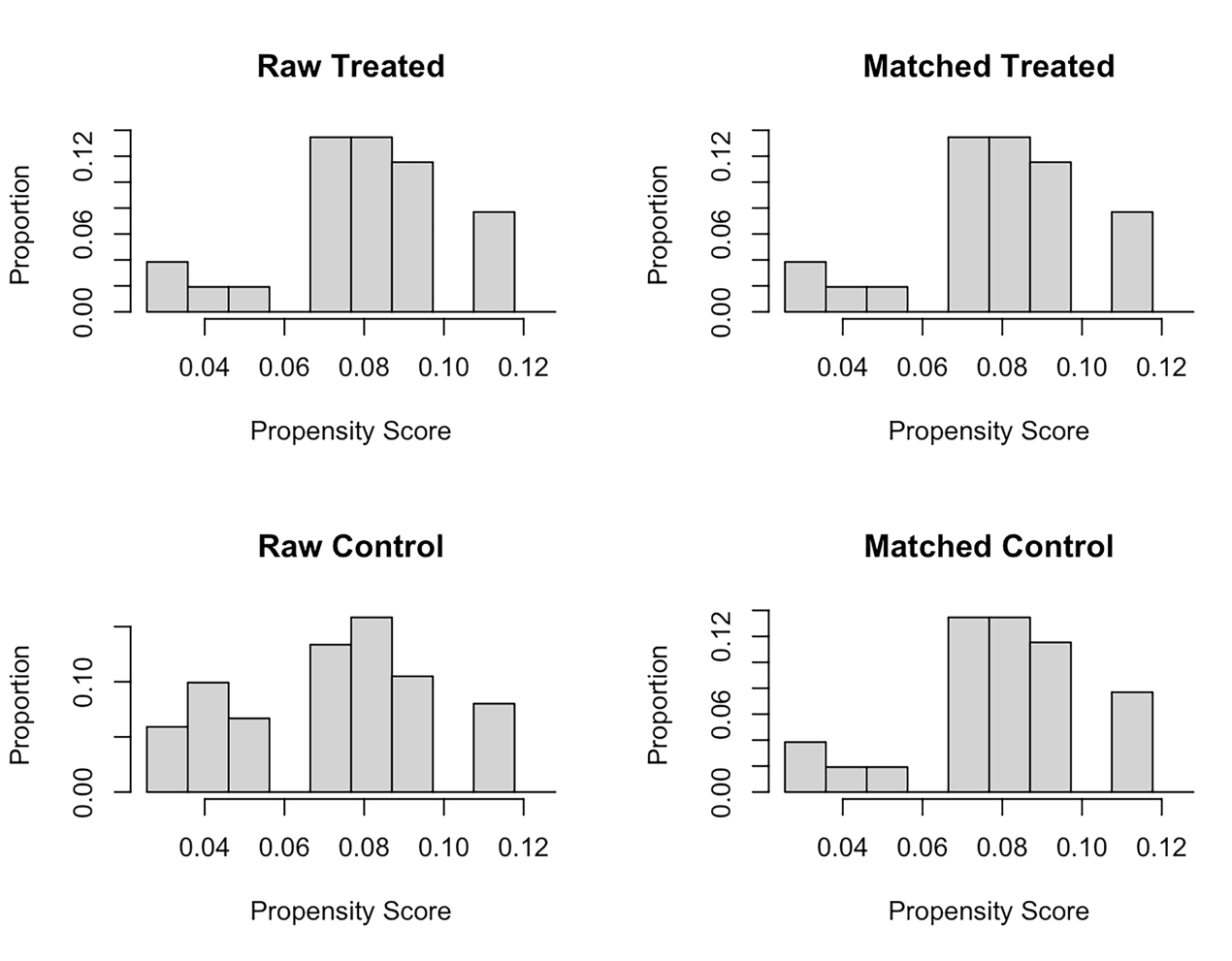


Figure S1


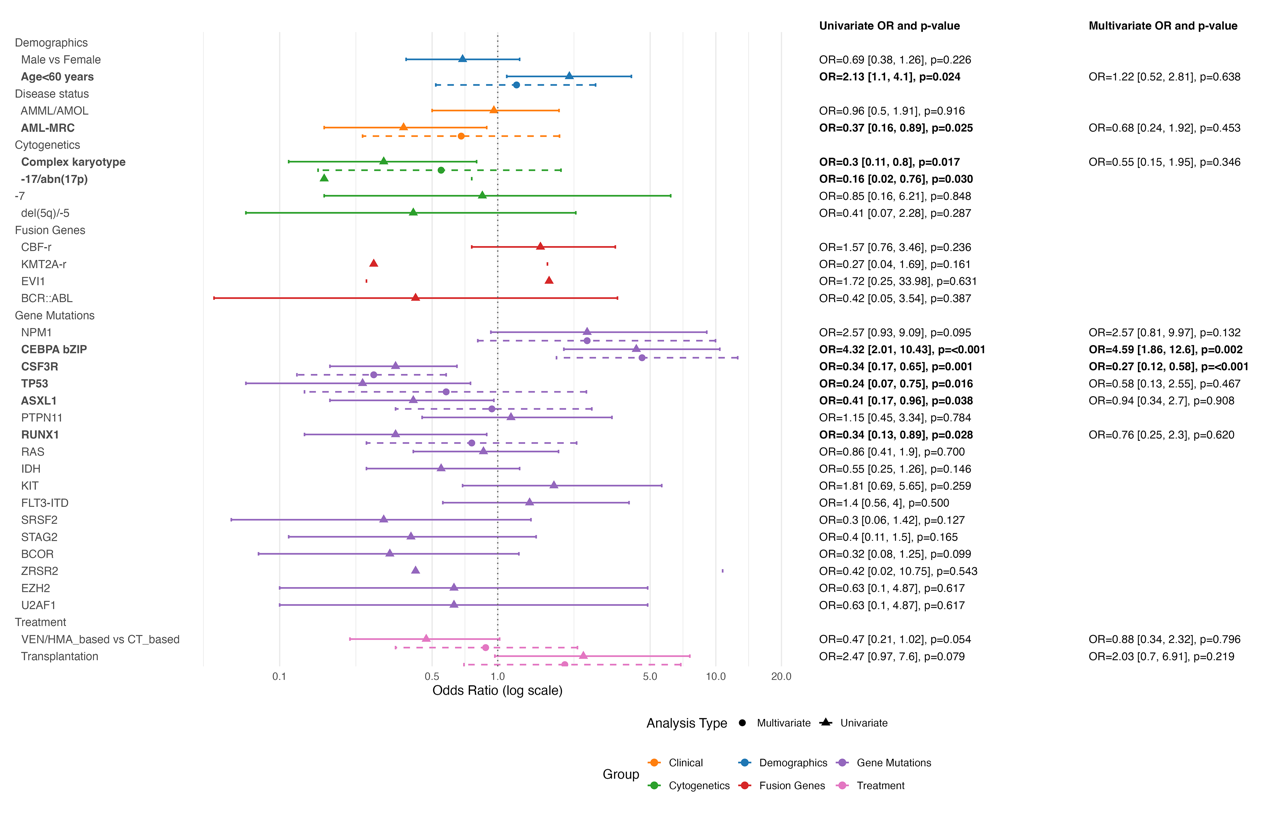


Figure S2


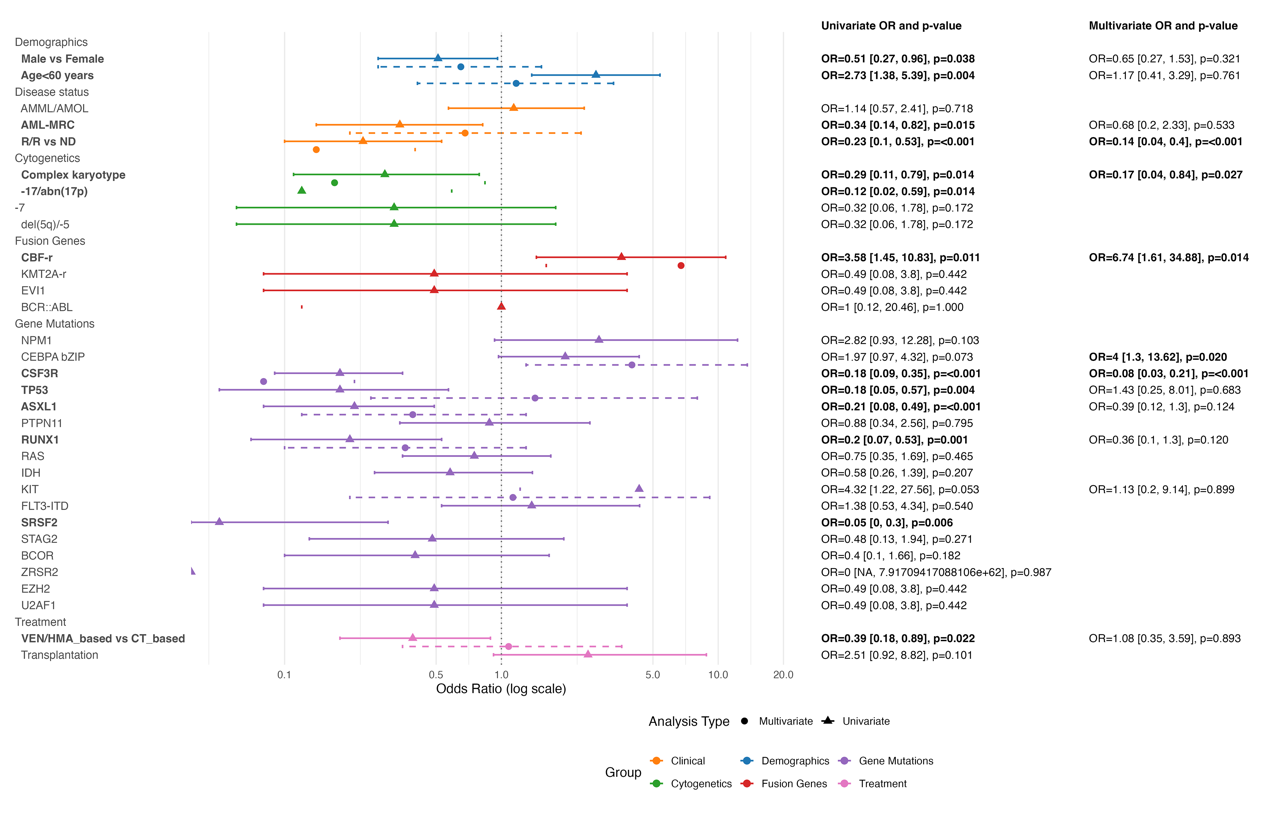
 Figure S3


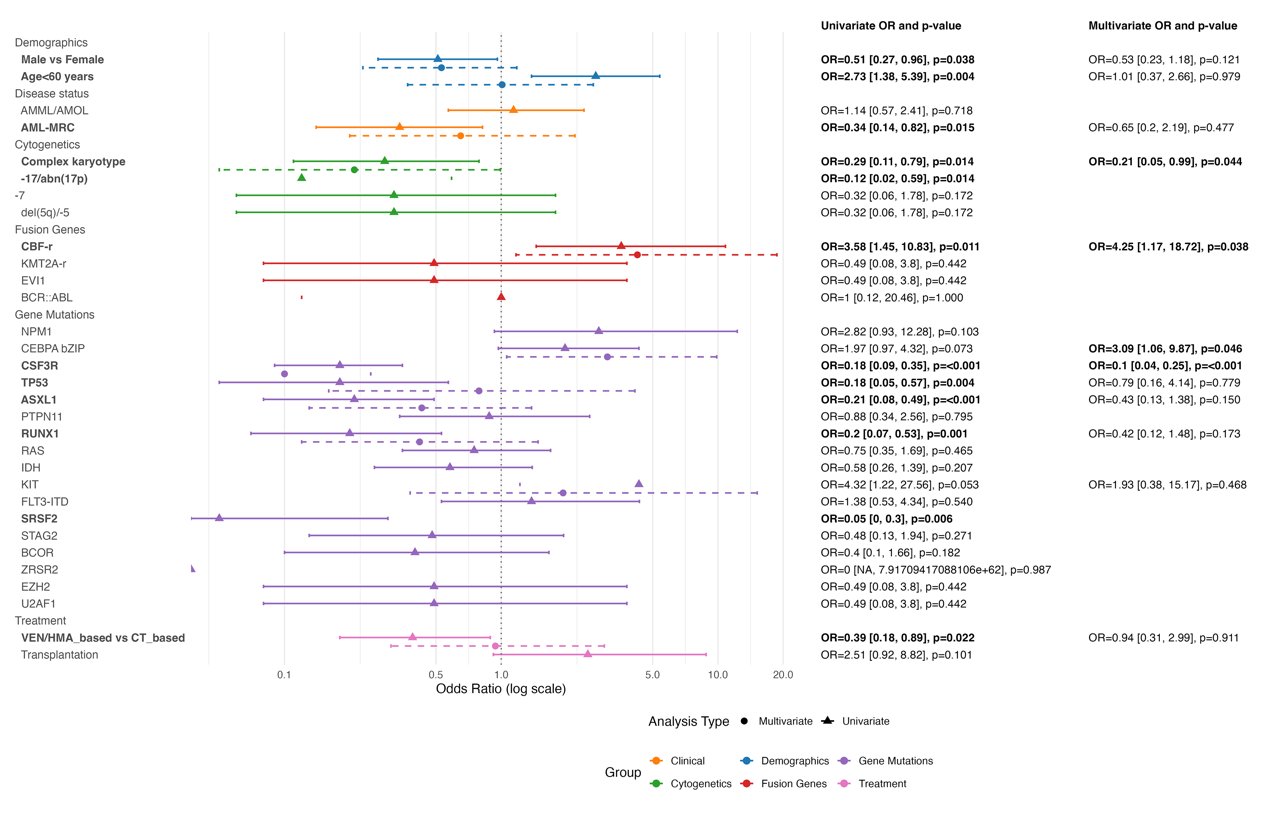


Figure S4
